# The arithmetic problem size effect in children: an event-related potential study

**DOI:** 10.3389/fnhum.2014.00756

**Published:** 2014-09-25

**Authors:** Leen Van Beek, Pol Ghesquièr, Bert De Smedt, Lieven Lagae

**Affiliations:** ^1^Parenting and Special Education Research Unit, Faculty of Psychology and Educational Sciences, University of LeuvenLeuven, Belgium; ^2^Department of Development and Regeneration, Biomedical Sciences Group, University of LeuvenLeuven, Belgium

**Keywords:** event-related potential (ERP), arithmetic, problem size effect, children, verbal production task

## Abstract

This study used for the first time event-related potentials (ERPs) to examine the well-known arithmetic problem size effect in children. The electrophysiological correlates of this problem size effect have been well documented in adults, but such information in children is lacking. In the present study, 22 typically developing 12-year-olds were asked to solve single-digit addition problems of small (sum ≤ 10) and large problem size (sum > 10) and to speak the solution into a voice key while ERPs were recorded. Children displayed similar early and late components compared to previous adult studies on the problem size effect. There was no effect of problem size on the early components P1, N1, and P2. The peak amplitude of the N2 component showed more negative potentials on left and right anterior electrodes for large additions compared to small additions, which might reflect differences in attentional and working memory resources between large and small problems. The mean amplitude of the late positivity component which follows the N2, was significantly larger for large than for small additions at right parieto-occipital electrodes, in line with previous adult data. The ERPs of the problem size effect during arithmetic might be a useful neural marker for future studies on fact retrieval impairments in children with mathematical difficulties.

## INTRODUCTION

Arithmetic skills are fundamental in our everyday life and represent an important part of the children’s curriculum at school. These skills have been extensively investigated with behavioral methods but more recently the use of neural measures, such as functional magnetic resonance neuroimaging or fMRI (see Arsalidou and Taylor, 2011 for a review of adult studies; see Kaufmann et al., 2011 for a review of children studies) and electrophysiology (e.g., adults: Núñez-Peña et al., 2011; children: Zhou et al., 2011), has provided evidence on the neurobiological basis of arithmetic processing. These neurobiological insights are particularly relevant for understanding the origins of atypical mathematical development or dyscalculia (Butterworth et al., 2011), an approach that has already been successful in the domain of dyslexia (Gabrieli, 2009). In sharp contrast to the number of fMRI studies about the neural correlates of arithmetic, only a limited number of studies in this field have used electrophysiological methods. However, electrophysiology is particularly relevant because it offers a higher temporal resolution and might be more child friendly than fMRI.

One of the most robust phenomena in the field of mathematical cognition is the problem size effect, which indicates that reaction time (RT) and error rate increase as the magnitude of the operands in an arithmetic problem increases (e.g., Stazyk et al., 1982; Campbell and Graham, 1985; Dehaene, 1992; Ashcraft and Guillaume, 2009). Numerous behavioral studies have reported this problem size effect in adults and children (see for a review Zbrodoff and Logan, 2005). The electrophysiological correlates of this problem size effect have been well documented in adults (Jost et al., 2004a,b; Núñez-Peña et al., 2005, 2006, 2011; Núñez-Peña, 2008), which makes this effect an excellent paradigm to investigate mental arithmetic. To the best of our knowledge, there are no studies that have examined this problem size effect in children. Against this background, the present study sets out to explore the electrophysiological correlates of the arithmetic problem size effect in children.

The problem size effect can be observed in all four basic arithmetic operations (addition, subtraction, multiplication, and division; e.g., Ashcraft and Battaglia, 1978; Campbell and Graham, 1985; LeFevre and Morris, 1999; Seyler et al., 2003) and has been obtained in both production and verification tasks (Parkman, 1972; Zbrodoff and Logan, 1990; Ashcraft, 1992; Campbell and Fugelsang, 2001). The problem size effect in adults is determined by different factors. First, strategic performance differences are significant sources of the problem size effect (e.g., LeFevre et al., 1996a; Campbell and Xue, 2001; see for a review: Zbrodoff and Logan, 2005). More specifically, the problem size effect is influenced by strategy selection and strategy efficiency. Strategy selection refers to the choice of a strategy among a set of available strategies (Imbo and Vandierendonck, 2008), often subdivided into direct memory retrieval and more procedural strategies such as counting (e.g., 8 + 3 = 9, 10, 11) and decomposition (e.g., 8 + 3 = 8 + 2 + 1 = 10 + 1 = 11). Memory retrieval is typically used more frequently on small than on large problems, and because retrieval is more efficient than procedure use, this explains the problem size effect. Strategy efficiency refers to how quickly and accurately strategies lead to the solution (Imbo and Vandierendonck, 2008). Both retrieval and procedural use are more efficient on small than on large problems, again leading to the problem size effect. Several studies have addressed the relationship between strategy selection and arithmetic skill and it has been reported that high-skilled individuals retrieve arithmetic facts more frequently and more efficiently than low-skilled individuals (LeFevre et al., 1996a; Imbo et al., 2007). Second, it has also been suggested that practice is an important determinant of the problem size effect (Pauli et al., 1994; Núñez-Peña, 2008). Small problems are more frequently processed than large problems and consequently small problems have a stronger memory trace and are therefore retrieved faster from long-term memory than large problems (Zbrodoff and Logan, 2005; Imbo and Vandierendonck, 2008; Grabner and De Smedt, 2011). In fact, practice can help to strengthen the problem-answer association and reduces the problem size effect. Current explanations of the problem size effect in children consider it to be driven by the same strategic performance differences as in adults (Barrouillet and Lepine, 2005; Imbo and Vandierendonck, 2008). More specifically, a smaller problem size effect has been associated with higher retrieval frequency and higher strategy efficiency (Imbo and Vandierendonck, 2008). Moreover, this strategy efficiency was related to individual differences in working memory span: low-span children executed both retrieval and procedural strategies less efficiently than high-span children. In line with these findings, Barrouillet and Lepine (2005) reported that children with lower working memory capacities exhibit a stronger problem size effect even when they only rely on retrieval, compared with children with high working memory capacities.

By recording event-related potentials (ERPs), previous electrophysiological studies have provided objective quantitative data on the temporal course of calculation. The solution of an arithmetic problem typically consists of three parts: encoding (i.e., converting a stimulus into appropriate internal codes), retrieving or calculating the answer, and responding (i.e., reporting the answer; Campbell, 1994; Campbell and Epp, 2005). Several ERP studies in adults have suggested that the early portion of the ERPs (i.e., up to around 250 ms post-stimulus) reflects physical identification of the stimuli (Iguchi and Hashimoto, 2000; El Yagoubi et al., 2003). The P1, N1, and P2 components typically occur at posterior electrodes within the first 250 ms post-stimulus. Studies in adults have reported no differences in these early posterior ERP components P1, N1, and P2 between small and large problems (Núñez-Peña et al., 2005), which indicates that the encoding is a similar mental process for small and large problems. After the encoding phase, a negativity between 300 and 500 ms with a maximum over anterior electrodes is usually observed, and this negativity, mostly referred to as N2 or N400, is larger for incorrect than for correct solutions in verification tasks (Niedeggen and Rösler, 1999; Niedeggen et al., 1999; Jost et al., 2004b; Szucs and Csepe, 2004, 2005; Zhou et al., 2006). The interpretation of this early anterior negativity has been highly debated. The component is sometimes interpreted as an index of mismatch processing, a reflection of the subject being surprised by the incorrect solution in verification tasks because it is elicited whenever a solution does not fit with the preceding equation (Niedeggen and Rösler, 1999; Niedeggen et al., 1999; Szucs and Csepe, 2004, 2005). This frontal negativity is probably not specific to calculation as it has been elicited in various tasks with diverse types of stimuli (for a review see Folstein and Van Petten, 2008). More specifically, the N2 with an anterior scalp distribution has been observed by using auditory as well as visual stimuli and in tasks such as verification, standard odd-ball and go/no-go paradigms that have been used to study, for example, arithmetic, reading, executive functioning, and working memory. On the other hand, this negativity around 400 ms post-stimulus is also thought to be related to differences in linguistic and working memory functions. In some adult studies, this component is interpreted as an “arithmetic” N400 similar to the classic “semantic” N400 (Niedeggen and Rösler, 1999; Niedeggen et al., 1999; Jost et al., 2004b; Zhou et al., 2006), which suggests the implication of verbal processing in arithmetic. Zhou et al. (2006) reported a smaller anterior negativity around 300 ms for addition than for multiplication, which might point to less phonological processing in addition than in multiplication. Furthermore, large problems are typically solved more often by procedural strategies, which require more working memory resources. The effect of problem size on this anterior negativity may therefore reflect differences in attentional and working memory resources, which are recruited more during large problems than during small problems. This also echoes data from fMRI studies, which show larger frontal activity in large than in small problems (e.g., Arsalidou and Taylor, 2011, for a review). To the best of our knowledge, only Jost et al. (2004b) investigated the problem size effect of the N2 component. They found that adults evoked relatively more negative potentials for large problems than for small problems between 360 and 780 ms and that the peak was reached later for large than for small problems. These authors suggested that the problem size effect was caused by both differences in the activation of the correct result and differences in solution strategies for small and large problems.

Event-related potentials during arithmetic in adults also revealed the existence of a late positive slow wave (e.g., Pauli et al., 1994, 1996; Niedeggen and Rösler, 1999; Iguchi and Hashimoto, 2000; El Yagoubi et al., 2003; Núñez-Peña et al., 2005, 2006; Szucs and Csepe, 2005; Núñez-Peña and Escera, 2007; Núñez-Peña, 2008; Prieto-Corona et al., 2010; Szucs and Soltesz, 2010; Chen et al., 2013). This late component, which shows a posterior distribution and starts at about 400 to 500 ms post-stimuli, may be the brain signature of the problem size effect. More specifically, the amplitude of this late positive slow wave increases as the problem size increases (Pauli et al., 1994, 1996; Núñez-Peña et al., 2005, 2006; Núñez-Peña, 2008). This amplitude modulation has been reported for multiplication (Pauli et al., 1994, 1996), addition and subtraction (Núñez-Peña et al., 2005, 2006). The amplitude of this positive slow wave is reduced by practice, probably because practice strengthens the memory trace and encourages the use of retrieval (Pauli et al., 1994; Núñez-Peña, 2008).

In sharp contrast to the number of ERP studies on arithmetic in adults, little is known about the neurophysiological correlates of arithmetic in children. To the best of our knowledge, only three studies have investigated this issue (Xuan et al., 2007; Prieto-Corona et al., 2010; Zhou et al., 2011). These studies, which compared the ERPs of adults and children during calculation tasks, indicate that similar to adults, children elicited an anterior negativity peaking around 400 ms post-stimulus and a subsequent late positive slow wave during arithmetical tasks. Despite these similarities, children displayed larger amplitudes, longer latencies, and a more widespread activation for these components than adults, probably due to greater cognitive effort. Importantly, it should be noted that none of the existing ERP children studies investigated the problem size effect.

Although several adult ERP studies have examined the effect of problem size, most of them have investigated this effect in a (delayed) verification task: the problem is presented first (either all terms of the arithmetic problem at once or each term sequentially), and after a specific time interval or together with the equation, a potential solution is presented. Participants have to evaluate whether the solution was correct or incorrect. Verification tasks have several disadvantages. First, verification tasks with sequential presentation of the arithmetic problem and solution generate two phases related to calculation (Chen et al., 2013): the production phase (between the offset of equations and the onset of potential solutions) and the verification/comparison phase (between the onset of potential solutions and the participant’s response). Some studies investigated the production phase by studying brain activity time-locked to the offset of equations (Núñez-Peña et al., 2011), whereas others investigated the verification phase by studying the brain activity time-locked to the proposed solutions of the problem (Niedeggen et al., 1999; Jost et al., 2004b; Szucs and Csepe, 2005; Luo et al., 2009; Szucs and Soltesz, 2010). Secondly, a growing number of studies have showed that mismatch processing in verification tasks based on for example the plausibility (Jost et al., 2004b; Núñez-Peña and Escera, 2007) and parity, i.e., whether the solution to a problem should be even or odd (Krueger and Hallford, 1984; Vandorpe et al., 2005), of solutions affect participants’ judgments and, consequently, the ERP waves. Taken together, this means that the specific calculation of the solution might take place either during the first phase (if the participants start to calculate as soon as the equation is presented) or during the second phase (if the participants do not start to calculate until the potential solution is present). But in addition to this, participants sometimes may not need to calculate the answer to a problem, because they can solve it by means of the easier and faster side-step strategies. For example, incorrect solutions might be rejected based on plausibility criteria, such as being mathematically very far from the correct solution (i.e., plausibility-checking strategy) or incorrect solutions might be rejected when the odd/even status of the proposed solution mismatches the correct answer (i.e., parity-checking strategy). The use of verification tasks therefore fails to capture the specific calculation process. We aimed to overcome this problem by using a production task, which guarantees that a participant really calculates the solution. This avoids the aforementioned mismatch effect. It is true that ERP studies typically avoid such verbal production tasks, because overt responses might produce movement artifacts in the EEG signal. However, we were primarily interested in the encoding and retrieval/calculation phase. To eliminate as much as possible motor-and speech artifacts related to the production of the answer, we only included EEG data from problem presentation until 125 ms before the fastest oral response, i.e., 800 ms post-stimulus. This approach has been successfully used in previous electrophysiological research of arithmetic (De Smedt et al., 2009; Grabner and De Smedt, 2011).

The present ERP study is the first in which the problem size effect was assessed in children using a verbal production task. We presented 22 typically developing 12-year-olds single-digit addition problems of small and large problem size, with small problems having sums ≤10 (e.g., 2 + 3) and large problems having sums >10 (e.g., 8 + 7). This categorization of small and large addition problems has been used in previous studies (e.g., LeFevre et al., 1996a; De Smedt et al., 2011). The children were instructed to solve the problem as quickly and accurately as possible. They had to speak the solution into a voice-key. Based on the adult literature reviewed above, we focused on the early components (P1, N1, and P2), the N2 component and the late positive slow wave in the ERP pattern. Firstly, as the early part of ERPs is considered to be a reflection of the identification of the stimulus, no differences between small and large problems were expected up to 250 ms post-stimulus, as the encoding of small and large problems was expected to be similar. Secondly, we expected to find an anterior negativity around 400 ms, with larger amplitudes for large than for small problem sizes. Finally, we focused on a late positive slow wave that emerges around 500 ms post-stimulus, the amplitude of which we predicted to be dependent on problem size, with smaller amplitudes for small problems and larger amplitudes for large problems.

## MATERIALS AND METHODS

### PARTICIPANTS

Twenty-two typically developing 12-year-old children participated in this study (*M =* 11.9 years; SD = 0.4; age range: 11.4–12.7 years; 11 boys; 17 right-handed). They all had normal intelligence (IQ > 88; *M =* 109; SD *=* 12) as determined by an abbreviated version of the Dutch Wechsler Intelligence Scale for Children, Third Edition (WISC-III-NL; Kort et al., 2005). All children had normal or corrected-to-normal vision. The parents of the children did not report any history of neurologic problems, psychiatric disorders or learning difficulties. Children were recruited from local schools. The study was approved by the local Medical Ethical Board of the university and written informed consent according to the Declaration of Helsinki was obtained from the children and their parents.

### STIMULI AND EXPERIMENTAL PROCEDURE

Single-digit addition problems of the form *a + b* were used as stimuli. The problems were selected from all possible pairwise combinations of the digits between 2 and 9, with the exclusion of tie problems (e.g., 4 + 4) and problems containing a 0 or 1 as operand or answer. These problems were excluded due to their unique encoding characteristics, an approach that has been used in previous studies in arithmetic (e.g., LeFevre et al., 1996b; Imbo and Vandierendonck, 2008; De Smedt et al., 2011). This set comprises 56 problems. From this set, 20 small (sums ≤ 10) en 20 large (sums > 10) problems were selected and each problem was presented twice. The position of the largest addend was counterbalanced for both problem types.

The experiment was executed by using Presentation software (Neurobehavioral Systems, Inc., Albany, CA, USA). Numbers were presented in white against a black background, and subtended a visual angle of 2.01° vertically and 5.27° horizontally. Arithmetic problems were presented on the screen and the participant was instructed to mentally solve the problem and subsequently speak the solution into a voice-key. Both accuracy and speed were stressed.

Following electrode placement and impedance calibration, the experimental procedure was described to the child. The child was seated comfortably in a dimly lit registration room and was instructed to avoid movements to reduce muscle artifacts in the EEG signal. The child had to look at the middle of the computer screen placed in front and to maintain fixation to avoid unnecessary eye movements. The instruction for the task was given immediately before the task. During the experiment, the experimenter sat out of sight of the child.

**FIGURE 1:**
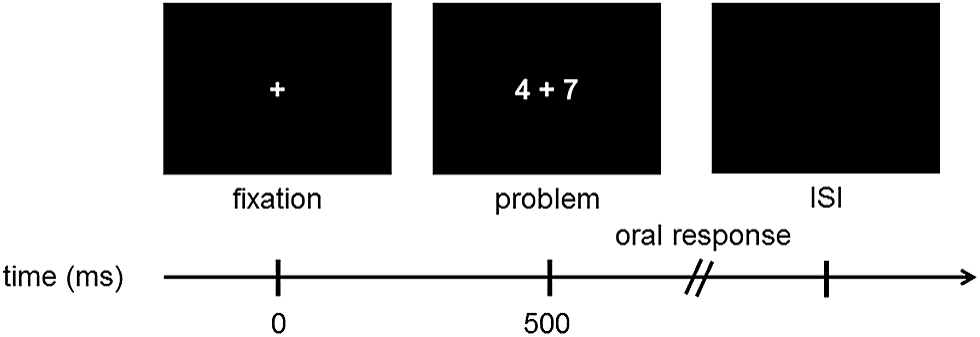
**Schematic display of one trial.** ISI, interstimulus interval of 1500 ms.

The child performed one practice run with 12 trials to ensure good understanding of the task and to prevent movements during the experimental task. More specifically, the children were trained in avoiding any movement during the mental calculation process that preceded the overt solution production. In addition, the children were trained in limiting articulatory movements during the actual production of the solution. Following the practice run, all participants were tested on 80 trials, which were organized into 4 runs of 20 trials separated by rest periods. The temporal sequence of one trial is depicted in **Figure 1**. Each trial consisted of (1) a fixation cross in the center of the screen which remained visible for 500 ms, (2) the addition problem which was shown until response or for a maximum 10,000 ms, and (3) a fixed interstimulus interval (ISI) of 1500 ms.

### ELECTROPHYSIOLOGICAL RECORDING

Electrode placement was done according to the international 10-10 system (Nuwer et al., 1998; Jurcak et al., 2007) with use of EEG recording cap with Ag/AgCl sintered ring electrodes (Easy Cap). Thirty-one electrodes were placed at Fp1, Fp2, F3, F4, F7, F8, Fz, FC1, FC2, FC5, FC6, FT9, FT10, C3, C4, Cz, CP1, CP2, CP5, CP6, T3, T4, T5, T6, P3, P4, Pz, PO9, PO10, O1, and O2. Additional four electro-oculogram (EOG) electrodes were placed resulting in two EOG channels: horizontal EOG – two electrodes on the outer canthi of eyes, and vertical EOG – two electrodes above and below one eye. EOG channels allowed us to detect both vertical and horizontal eye movements and to effectively remove these eye movements from EEG recording during subsequent preprocessing of the signal (see below). Two linked mastoid electrodes were used as a reference. EEG was sampled at a frequency of 1000 Hz with 12 bits A/D converter and amplified using a band-pass filter of 70 Hz. Registration of the digital EEG was made using the software program BrainRT (OSG, Belgium). The impedance of all electrodes was monitored for each participant prior to recording and was always kept below 5 kΩ.

### DATA ANALYSIS

#### Behavioral data

Mean error rate (percentage of incorrect responses) and mean RT for correctly solved trials were analyzed with a three-way repeated measures analysis of variances (ANOVAs), taking Problem size (small vs. large) as within-subject factor. P-values were corrected by Greenhouse–Geisser correction for sphericity departures when appropriate.

#### EEG analysis

Data processing was performed offline using the EEGLAB vs.10.2 toolbox (Matlab R2008a platform; Delorme and Makeig, 2004). During preprocessing, data were filtered with a 30 Hz digital low pass filter. Eye movement artifacts were marked and removed from the continuous signal without affecting the signal itself with an algorithm based on the principle of Independent Component Analysis (Hyvarinen and Oja, 2000; Mennes et al., 2010). EEG fragments that contained other movement artifacts were removed based on visual inspection of the data. After preprocessing, the continuous EEG signal was epoched including a 200 ms pre-stimulus baseline period and a 900 ms post-stimulus period. Next, epochs for every participant in each experimental condition were averaged and incorrect trials and trials with artifacts (voltage exceeded ± 120 μV in any electrode site) were excluded.

ERPs were time-locked to the onset of the arithmetic problems and were quantified as peak amplitudes and latencies in the 100–150 (P1 component), 150–250 (N1 component), 150–250 (P2 component) and 250–500 (N2 component) milliseconds windows following the arithmetic stimuli. The time windows of these early components were based on the grand mean waveforms and previous ERP research in arithmetic in children (Xuan et al., 2007; Zhou et al., 2011) and adults (Núñez-Peña et al., 2005; Núñez-Peña and Escera, 2007; Luo et al., 2009; Prieto-Corona et al., 2010; He et al., 2011). The late slow wave, i.e., late positivity component (LPC), which has been put forward as a brain signature of the problem size effect, was defined as having a mean amplitude value in the 500–675 ms range. This time window was chosen because it is the window where the LPC has been described in previous ERP research in adults (Niedeggen and Rösler, 1999; Núñez-Peña et al., 2005, 2006, 2011; Núñez-Peña and Escera, 2007; Núñez-Peña, 2008) and children (Prieto-Corona et al., 2010). We only analyzed the mean amplitude of the LPC, and not the peak amplitude or peak latency of the LPC because the LPC is a slow wave component without a clear starting point, peak, and ending point. Therefore we analyzed the mean amplitude over a time window where the LPC has been described in previous research. This approach has been used previous studies on the LPC (e.g., Niedeggen and Rösler, 1999; Núñez-Peña et al., 2006, 2011; Núñez-Peña and Escera, 2007). Data later than 125 ms before the first oral response of any child as registered by the voice key (i.e., 800 ms post-stimulus) were not included to account for the delay of the voice-key trigger signal and to eliminate motor- and speech-related artifacts when producing the answer into the voice-key. This approach has been successfully used in previous electrophysiological research during arithmetic (De Smedt et al., 2009; Grabner and De Smedt, 2011).

The early components P1, N1, and P2 were analyzed at the following posterior electrode sites: C3, Cz, C4, CP5, CP1, CP2, CP6, P3, Pz, P4, PO9, PO10, O1, and O2. The selection of electrode sites was based on the existing body of evidence (e.g., Núñez-Peña et al., 2005; Zhou et al., 2011) and visual inspection of the data. For statistical analyses, ERPs were aggregated over five cortical areas per hemisphere: central left (C3), central right (C4), centro-parietal left (CP5, CP1), centro-parietal right (CP2, CP6), parietal left (P3), parietal right (P4), parieto-occipital left (PO9), parieto-occipital right (PO10), occipital left (O1), occipital right (O2). Peak latencies and amplitudes of these early components were analyzed using ANOVA, taking problem size (small vs. large), caudality (central vs. centro-parietal vs. parietal vs. parieto-occipital vs. occipital), and hemisphere (left vs. right) as within-subject factors. Midline sites, i.e., Cz and Pz, were analyzed separately. For these midline sites, a two-way repeated measures ANOVA was carried out with problem size (small vs. large) and caudality (central vs. parietal) as within-subject factors.

The following electrode sites (Fp1, Fp2, F7, F3, Fz, F4, F8, FC5, FC1, FC2, FC6, C3, Cz, and C4) were selected for statistical analysis of the N2 component. The analysis of this N2 component was restricted to these electrodes based on inspection of the data and because the early negativity component is known to have regularly an anterior maximum (e.g., Szucs and Csepe, 2005; Zhou et al., 2006, 2011; Xuan et al., 2007; Luo et al., 2009). For statistical analyses, ERPs were aggregated over four cortical areas per hemisphere: prefrontal left (Fp1), prefrontal right (Fp2), frontal left (F7, F3), frontal right (F4, F8), fronto-central left (FC5, FC1), fronto-central right (FC2, FC6), central left (C3), and central right (C4). Peak latencies and amplitudes of this N2 component were analyzed using a three-way repeated measures ANOVA, taking problem size (small vs. large), caudality (prefrontal vs. frontal vs. fronto-central vs. central), and hemisphere (left vs. right) as within-subject factors. Midline sites, i.e., Fz and Cz, were analyzed separately. For these midline sites, a two-way repeated measures ANOVA was carried out with problem size (small vs. large) and caudality (frontal vs. central) as within-subject factors.

The LPC was analyzed at the following electrode sites: P3, Pz, P4, PO9, PO10, O1, and O2. Statistical analyses were performed over three areas per hemisphere: parietal left (P3), parietal right (P4), parieto-occipital left (PO9), parieto-occipital right (PO10), occipital left (O1), and occipital right (O2).The mean amplitudes of this LPC were analyzed using a three-way repeated measures ANOVA, with problem size (small vs. large), caudality (parietal vs. parieto-occiptal vs. occipital), and hemisphere (left vs. right) as within-subject factors. The midline site Pz was analyzed separately. For this electrode site, a one-way ANOVA was carried out with problem size (small vs. large) as within-subject factor.

For all the statistical analyses the F value, the uncorrected degrees for freedom and probability level are reported. We used the Bonferroni correction for multiple comparisons where appropriate.

## RESULTS

### BEHAVIORAL DATA

The children solved small additions within 803–1531 ms (*M =* 1129 ± 212 ms) with an error rate of 0–5% (*M* = 3.14 ± 0.97%), whereas large additions were solved within 1045–2708 ms (*M* = 1707 ± 0 415 ms) with an error rate of 0–22.5% (*M* = 7.73 ± 0.94%). With regard to RT, there was a significant effect of problem size [*F*(1,24) = 91.37, *p* < 0.0001], showing that small problems were solved faster than large problems. Turning to error rate, there was a significant effect of problem size [*F*(1,24) = 14.27, *p* < 0.01], showing that fewer errors were made on small problems than on large problems.

### EVENT-RELATED POTENTIALS

#### Early components P1, N1, P2

**FIGURE 2:**
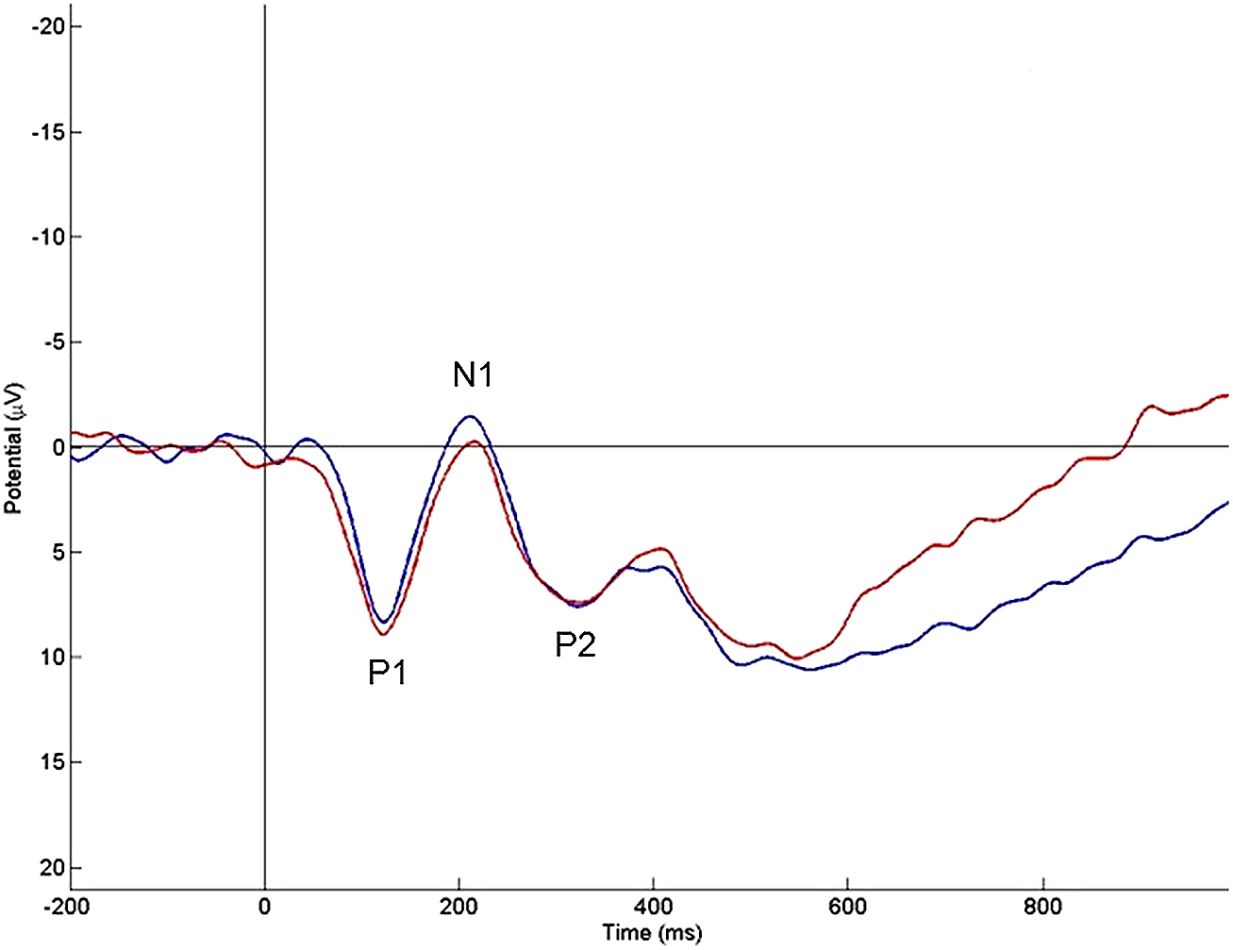
**The mean event-related potentials elicited by single-digit additions over representative electrode O2 (blue line, large problems; red line = small problems).** No differences among problem size in the early components P1, N1, and P2.

As expected, no differences between small and large problem size were found up to approximately 250 ms post-stimulus (see **Figure 2**). More specifically there was no significant main effect of problem size for P1 peak amplitude (*p* = 0.973), P1 peak latency (*p* = 0.678), N1 peak amplitude (*p* = 0.145) or N1 peak latency (*p* = 0.079). On P2, there was no main effect of problem size for the peak amplitude (*p* = 0.191), nor for the peak latency (*p* = 0.559).

#### N2 effect

**FIGURE 3:**
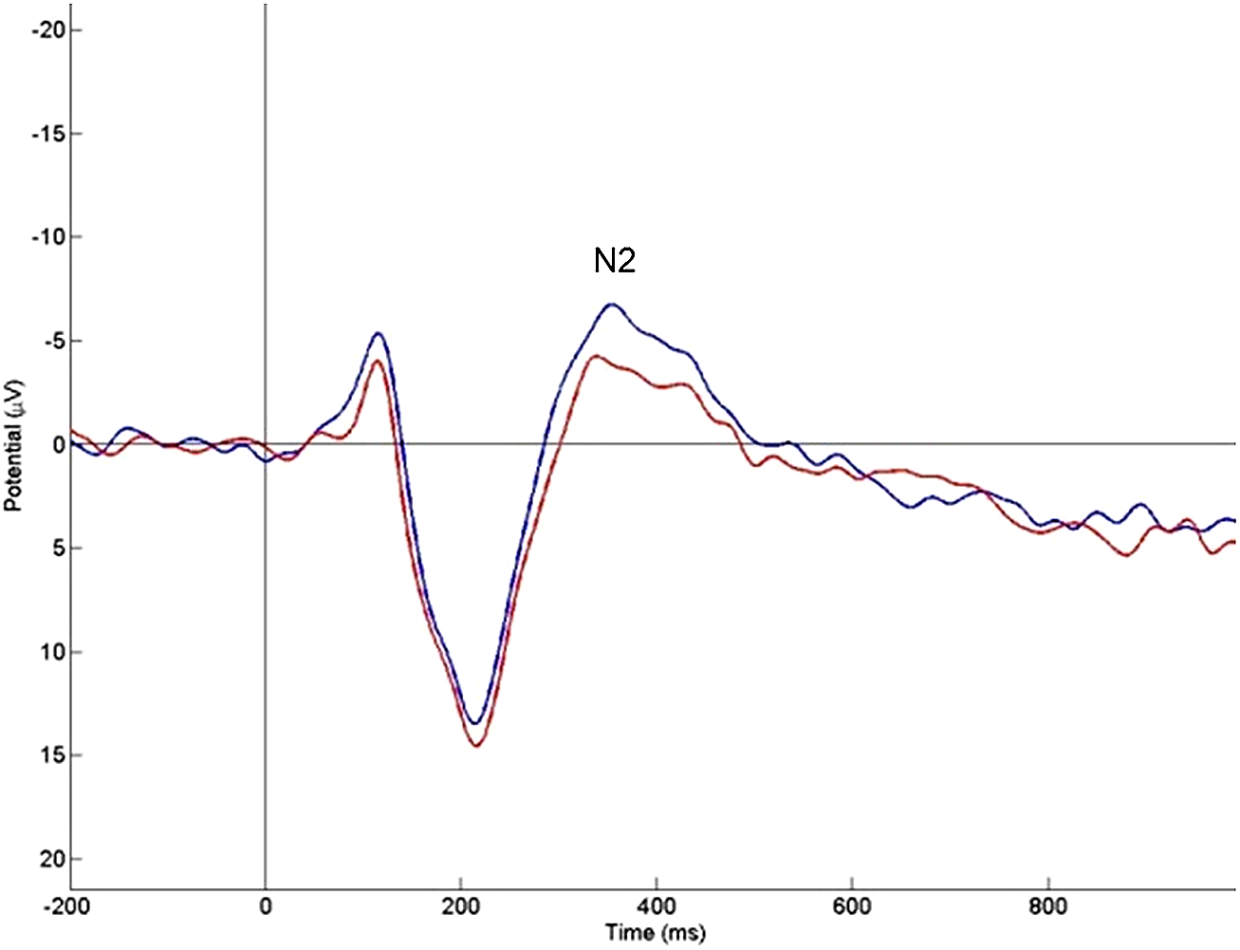
**The mean event-related potentials elicited by single-digit additions over representative electrode Fz (blue line, large problems; red line, small problems).** A significant problem size effect of amplitude can be observed in the N2 component around 400 ms.

The overall ANOVA for N2 peak amplitude revealed a significant main effect of problem size [*F*(1,21) = 0 12.57, *p* = 0.002]. *Post hoc* comparisons with Bonferroni adjustments revealed that large problems, when compared to small problems, elicited more negative potentials at the anterior electrodes over the scalp peaking around 400 ms, i.e., –6.39 μV versus –3.76 μV. Pairwise comparisons showed significant problem size effects at the prefrontal left area [*t*(21 = –2.51; *p* = 0.021], prefrontal right [*t*(21) = –2.48; *p* = 0.022], frontal left [*t*(21) = –3.32; *p* = 0.003], frontal right [*t*(21) = –2.28; *p* = 0.033], fronto-central left [*t*(21) = –2.51; *p* = 0.001], fronto-central right [*t*(21) = –2.30; *p* = 0.032], and central left [*t*(21) = –2.94; *p* = 0.008]. No significant difference was found at the central right area (*p* > 0.05). N2 peak amplitudes also showed significant main effects of problem size [*F*(3,63) = 6.25, *p* = 0.022] and caudality [*F*(3,63) = 54.86, *p* < 0.0001] in midline regions. The problem size only reached significance at Fz [*t*(21) = –2.53; *p* = 0.019; see **Figure 3**] and not at Cz (*p* > 0.05). The N2 amplitude was significantly more negative at Fz (–9.08 ± 1.36 μV) than at Cz (–1.63 ± 1.53 μV) and again large problems elicited larger amplitudes than small problems. The significant problem size effect (large minus small) in the peak amplitude of the N2 component varied between –1.79 and –2.62 μV depending on the topographical area (see **Table 1**). In addition to the significant problem size effect, the overall ANOVA for N2 peak amplitude revealed a significant main effect of caudality [*F*(3,63) = 49.41, *p* < 0.0001] as well. *Post hoc* comparisons with Bonferroni adjustments revealed that the N2 peak amplitude was significantly more negative at prefrontal (–8.95 ± 1.36 μV) than at frontal (–7.24 ± 1.32 μV) than fronto-central (–3.76 ± 1.19 μV) and central (–0.35 ± 1.10 μV) electrode sites. There was no effect of hemisphere (*p* > 0.05).

**Table 1:** The effect of problem size on the amplitude of the N2 component.

Topographical area	Problem size effect (μV, large minus small)
Prefrontal left	–2.12*
Prefrontal right	–2.12*
Frontal left	–2.38**
Frontal midline	–2.69*
Frontal right	–2.07*
Fronto-central left	–2.62**
Fronto-central right	–1.79*
Central left	–2.33**
Central midline	–
Central right	–

*p < 0.05; **p < 0.01.

*Only significant problem size effects are shown.*

Turning to N2 latency, the overall ANOVA showed no effect for problem size (*p* = 0.709) or hemisphere (*p* = 0.342), but a significant main effect of caudality [*F*(3,63) = 4.43, *p* = 0.008]. *Post hoc* comparisons with Bonferroni adjustments revealed that the N2 peak latency was significantly lower at prefrontal (358.76 ± 9.65 ms) than at frontal (365.64 ± 9.30 ms) than fronto-central (373.04 ± 9.62 ms) and central (373.04 ± 8.24 ms) electrode sites. No significant differences were found for midline regions.

#### LPC effect

**FIGURE 4:**
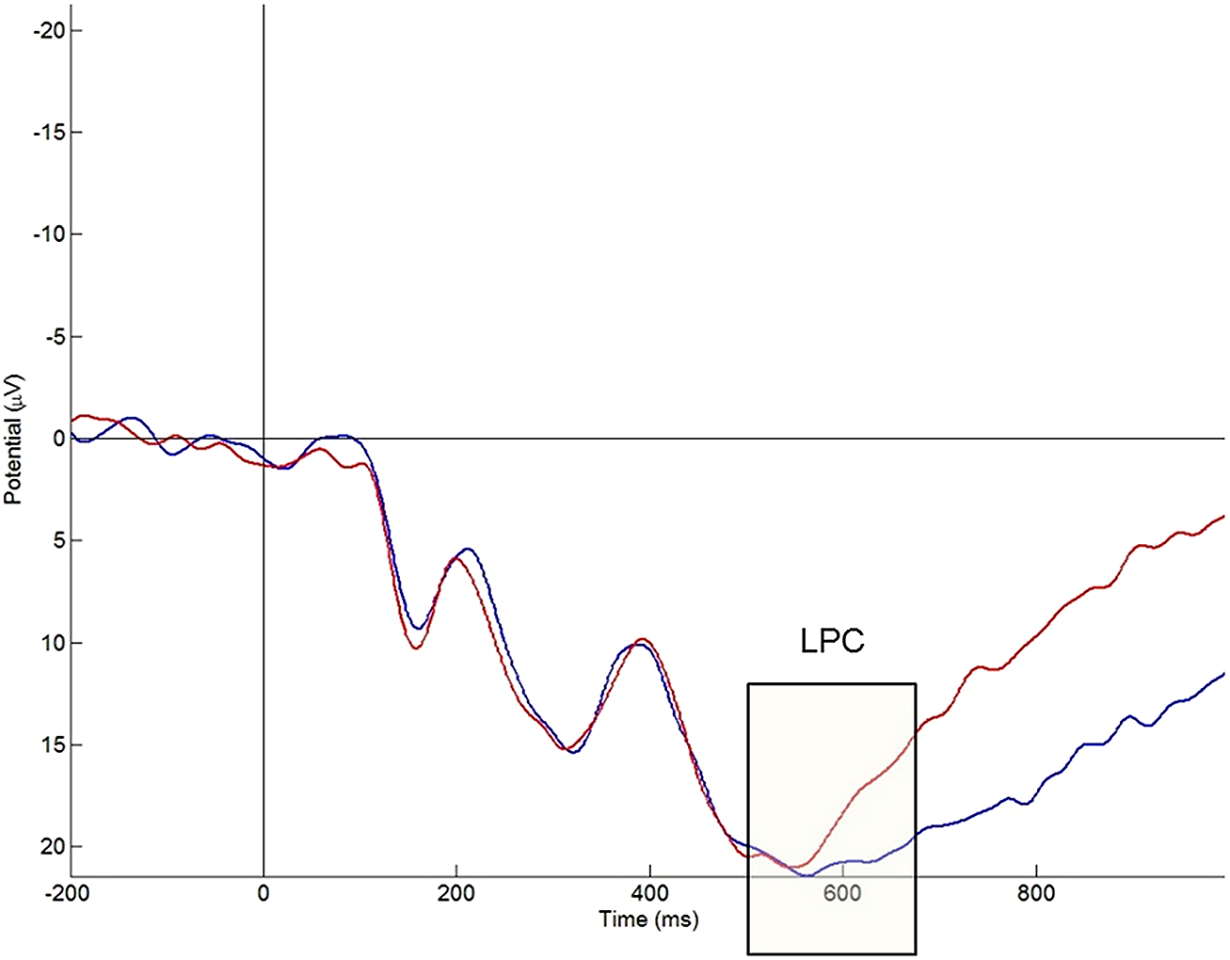
**The mean event-related potentials elicited by single-digit additions over representative electrode Pz (blue line, large problems; red line, small problems).** A significant problem size effect can be observed in the mean amplitude of the late positivity component (LPC) component.

The overall ANOVA for the mean amplitude of the LPC revealed a significant main effect of problem size [*F*(1,21) = 5.85, *p* = 0.025]. *Post hoc* comparisons with Bonferroni adjustments revealed that large problems had larger mean amplitudes in the 500–625 ms range compared with small problems, i.e., 7.91 ± 0.92 μV vs. 6.44 ± 0.084 μV. Pairwise comparisons showed significant problem size effects at the parietal right area [*t*(21) = 2.32; *p* = 0.030], parieto-occipital right [*t*(21) = 2.45; *p* = 0.023], occipital left [*t*(21) = 2.12; *p* = 0.046], and occipital right area [*t*(21) = 3.00; *p* = 0.007]. No significant differences were found in the left parietal area (*p* = 0.95) and left parieto-occipital area (*p* = 0.151). An effect of problem size [*F*(1,21) = 106.41, *p* < 0.0001] was significant at the midline electrode Pz (see **Figure 4**). Again large problems had larger mean amplitudes than small problems. Depending on the topographical area, the problem size-effect (large minus small) varied between 1.68 and 2.65 μV (see **Table 2**). The overall ANOVA for the mean amplitude of the LPC revealed significant effects of caudality [*F*(2,42) = 32.03, *p* < 0.0001] and hemisphere [*F*(1,21) = 5.66, *p* = 0.027]. *Post hoc* comparisons with Bonferroni adjustments revealed that the mean amplitude in the 500–625 ms range was higher in the right hemisphere (7.98 ± 0.93 μV) than in the left hemisphere (6.36 ± 0.86 μV) and that the mean amplitude was higher at parietal (11.63 ± 1.36 μV) than at occipital (7.95 ± 1.13 μV) and at parieto-occipital (1.93 ± 0.65 μV) electrode sites.

**Table 2:** Mean amplitude differences (in μV) between small and large problems in the 500–675 ms window.

Topographical area	Problem size effect (μV, large minus small)
Parietal left	–
Parietal midline	2.65*
Parietal right	1.68*
Parieto-occipital left	–
Parieto-occipital right	1.96*
Occipital left	1.74*
Occipital right	2.57**

*p < 0.05; **p < 0.01.

*Only significant problem size effects are shown.*

The overall ANOVA for the mean amplitude of the LPC revealed an interaction between problem size and hemisphere [*F*(1,21) = 8.47, *p* = 0.008]. Follow-up analyses revealed that the problem size effect was observed in the right [*F*(1,21) = 10.00, *p* = 0.005] but not in the left hemisphere.

The overall ANOVA for the mean amplitude of the LPC also revealed an interaction of problem size and caudality [*F*(1,21) = 3.58, *p* = 0.047]. A more detailed analysis of this interaction effect was performed by using separate ANOVAs at each caudality. The effect of problem size was only significant at parieto-occipital [*F*(1,21) = 4.62, *p* = 0.043] and occipital electrode sites [*F*(1,21) = 6.94, *p* = 0.015].

## DISCUSSION

Most of the existing electrophysiological studies on mental arithmetic have dealt with adult participants (e.g., Pauli et al., 1994, 1996; Iguchi and Hashimoto, 2000; Jost et al., 2004a,b; Núñez-Peña et al., 2005, 2006, 2011; Núñez-Peña and Escera, 2007; Núñez-Peña, 2008; Jasinski and Coch, 2012; Chen et al., 2013) while only a few of them have focused on children (Xuan et al., 2007; Prieto-Corona et al., 2010; Zhou et al., 2011). Extending this body of data, the present study is the first to use ERPs to investigate the arithmetic problem size effect, which is one of the most robust effects in the field of mathematical cognition. Although this effect has been investigated in adults (Jost et al., 2004b; Núñez-Peña et al., 2005, 2006, 2011; Núñez-Peña, 2008), there are no studies that examined this issue in children. Such research is relevant because knowledge about the electrophysiological correlates of the problem size effect in typically developing children might be useful for future studies in children with mathematical difficulties, particularly in view of the large individual differences in arithmetic strategy use in children (e.g., Dowker, 2005). Therefore, the main aim of the present study was to examine the ERPs elicited by small and large arithmetic problems in children.

The behavioral data of the current study showed clear problem size effects both on RTs and error rates, i.e., slower and less accurate performance on large problems than on small problems (see Zbrodoff and Logan, 2005 for a review). This observation is in line with previous behavioral research in children of a similar age (Barrouillet and Lepine, 2005; Imbo and Vandierendonck, 2008).

No differences between small and large problem sizes were found up to approximately 250 ms post-stimulus, i.e., in the P1, N1, and P2 component. This finding replicated previous ERP studies with verification tasks in adults (Iguchi and Hashimoto, 2000; El Yagoubi et al., 2003; Núñez-Peña et al., 2005), in which these early components were associated with the identification of the stimulus. This is also in line with the classical ERP pattern connected to visual stimuli recognition in adults (Simson et al., 1985; Czigler and Csibra, 1990). Only Zhou et al. (2011) investigated some of these early components, namely P1 and N1, during arithmetic in children and suggested that these early components likely reflect low-level processing rather than arithmetic processing. It should be noted that although Zhou et al. (2011) administered small and large addition problems, the authors did not analyze potential differences in the ERPs between these small and large additions. The present study adds new information to the findings of Zhou et al. (2011) by showing the existence of similar low-level processes for small problems and large problems.

With respect to the N2 component, our results show that large additions, compared to small additions elicit more negative potentials on the anterior electrodes over the scalp between 250 and 500 ms in both hemispheres. This finding is consistent with the observation of Jost et al. (2004b) who also found that larger problems evoked a relatively more negative potential than smaller problems. This observation might reflect the use of more attentional resources and working memory when solving large relative to small single-digit additions. This is also consistent with fMRI data collected during calculation tasks in children (De Smedt et al., 2011) and adults (e.g., Zhou et al., 2007; Jost et al., 2009), which showed higher activity over frontal areas when solving large problems. These researchers explained this observation by the use of more attentional and working memory resources when solving large problems compared to small problems. Likewise, differences in anterior negativity may reflect differences in load on working memory and control functions. This interpretation is in line with previous ERP findings of Prieto-Corona et al. (2010) and Xuan et al. (2007), who found greater N400 amplitudes in children than adults and explained this by the fact that children may exert greater effort when solving arithmetic problems. Similarly, our results may reflect slower and more effortful calculation for large problems than for small problems. We would like to point out that some authors have suggested that the left negativity around 400 ms observed in their ERP studies is an index of phonological processing (Niedeggen and Rösler, 1999; Niedeggen et al., 1999; Zhou et al., 2006, 2011; Luo et al., 2009). These adult studies have indicated that the left negativity is associated with verbal processing in arithmetic because phonological representations might be important for retrieval of existing arithmetic facts. Our pattern of N2 findings does not fit this interpretation. From this point of view, one would predict larger amplitudes for small problems than for large problems, because small problems are expected to be solved more with retrieval of verbally stored arithmetic facts. However, we observed the opposite effect. This seems to suggest that in the current study the effect of problem size on the N2 component reflects differences in working memory load and executive processes rather than phonological processing.

Prior adult ERP studies on arithmetic have reported that the negativity around 400 ms is followed by an LPC with posterior distribution (e.g., Niedeggen et al., 1999; Szucs and Csepe, 2005; Núñez-Peña, 2008) and an amplitude that is modulated by problem size (Pauli et al., 1994, 1996; Núñez-Peña et al., 2005, 2006; Núñez-Peña, 2008). Previous investigations of the LPC component in children are scarce. Only one ERP study in children examined the LPC in children during arithmetic (Prieto-Corona et al., 2010). In this study, children displayed an LPC in arithmetic verification tasks, but only for correct solutions. However, it is unclear whether this LPC is modulated by problem size, as in adults (Pauli et al., 1994, 1996; Núñez-Peña et al., 2005, 2006; Núñez-Peña, 2008). The children in the present study showed larger mean amplitudes of the LPC for large additions than for small additions at right parieto-occipital electrodes. This observation is in line with previous ERP studies in adults about the problem size effect (Pauli et al., 1994, 1996; Núñez-Peña et al., 2005, 2006; Núñez-Peña, 2008) that observed an increase in the mean amplitude of the LPC with problem size. Previous adult research explained the problem size effect for the LPC by the differences in frequency of exposure between small and large problems together with the use of different strategies (Núñez-Peña, 2008). More specifically, small problems are processed more frequently than large problems and therefore have a stronger problem-answer association in long-term memory, which means that they can be solved quickly by retrieval. On the other hand, larger problems are more often solved by slow procedures, such as counting and decomposition, and the answer is not directly retrieved from long-term memory.

We observed a right lateralized problem size effect on the LPC. To the best of our knowledge, no previous studies on the problem size effect on the LPC explored hemispherical differences, except for Núñez-Peña (2008). They found a somewhat right lateralized problem size effect. More specifically, the problem size effect reached only statistical significance at L2, L3, L4, and L5 when laterality was subdivided into five levels from left to right. This observation is in line with the right lateralized problem size effect in the current study. Our finding is also consistent with previous fMRI research that found greater activity for large than for small problems at right posterior brain areas, such as the right IPS (e.g., Stanescu-Cosson et al., 2000; Prado et al., 2013). The right lateralization of the LPC effect at posterior electrode sites might thus have originated from right posterior brain regions which are involved in visuospatial working memory and spatial attention (Corbetta et al., 2000; Diwadkar et al., 2000; Linden et al., 2003; Postle et al., 2004). In other words, the larger mean amplitude of the LPC at right posterior electrodes for large problems than for small problems might suggest that large problems involved more visuospatial processing to support the manipulation of numerical magnitudes.

Different from most of the existing ERP studies in arithmetic (e.g., Jost et al., 2004a,b; Núñez-Peña et al., 2005, 2006, 2011; Núñez-Peña and Escera, 2007; Núñez-Peña, 2008; Prieto-Corona et al., 2010; Zhou et al., 2011), the present study used a production and not a verification task to examine the electrophysiological correlates of arithmetic. This was done because verification tasks might fail to capture the specific calculation process. Indeed, in these verification tasks, multiple numerical and non-numerical processes can contribute to task execution. As mentioned in the introduction, participants may not need to calculate the problem to give their answer, because they can solve the problem by means of easier and faster side-step strategies such as the plausibility-checking strategy (Zbrodoff and Logan, 1990; Campbell and Tarling, 1996; Núñez-Peña and Escera, 2007). It is true that production tasks are often avoided in ERP-research, because they might increase the occurrence of movement artifacts that distort the EEG-signal. However, as we have described above, we have tried to avoid as much as possible such movement artifacts by thorough training of the children and by only analyzing the EEG-signal from stimulus presentation until 125 ms before the first verbal response of any child. The current study showed similar early and late components on ERPs during arithmetic by using a verbal-production task. To the best of our knowledge, this is the first ERP study that uses a verbal production paradigm to compare small and large problems. This is of particular interest because production tasks are more ecologically valid measures of mathematical performance than verification tasks. Indeed, verification tasks are rarely used in real-world classroom situations.

A growing body of evidence points to deficits in arithmetic fact retrieval in children with atypical mathematical development or dyscalculia (e.g., Jordan et al., 2003; Geary, 2004, 2010). The underlying causes of these deficits are largely unknown but structural (e.g., Isaacs et al., 2001; Rotzer et al., 2008) and functional (e.g., Price et al., 2007; Kucian et al., 2011) abnormalities in the brain, in particular in the inferior parietal cortex, have been observed (e.g., Butterworth et al., 2011). Developmental studies on brain activity during arithmetic have the potential to unravel the biological origin of dyscalculia and in the long run, these studies might lead to neural makers for detection of this disorder. This approach has already been successful in the domain of dyslexia (see for a review: Habib, 2000; Heim and Keil, 2004; Lyytinen et al., 2005), where ERPs have been shown to be effective indices of difficulties in auditory processing in dyslexia and ERPs to speech sounds in infants predict (impairments in) their reading development 8 years later (Molfese, 2000). Similarly, ERPs following arithmetic might be a neural marker of subsequent mathematical difficulties. Future research should investigate how the electrophysiological problem size effect differs between typically developing children and children with dyscalculia. Such research should also investigate whether training of arithmetic fact retrieval in children with dyscalculia has an impact on the brain signatures of their problem size effect. The ERP problem size design of the current study provides an excellent paradigm to probe such outstanding questions.

## Conflict of Interest Statement

The authors declare that the research was conducted in the absence of any commercial or financial relationships that could be construed as a potential conflict of interest.
